# Protective Effect of the Total Flavonoids from *Rosa laevigata* Michx Fruit on Renal Ischemia-Reperfusion Injury through Suppression of Oxidative Stress and Inflammation

**DOI:** 10.3390/molecules21070952

**Published:** 2016-07-21

**Authors:** Lisha Zhao, Lina Xu, Xufeng Tao, Xu Han, Lianhong Yin, Yan Qi, Jinyong Peng

**Affiliations:** College of Pharmacy, Dalian Medical University, Western 9 Lvshunnan Road, Dalian 116044, China; zhaolisha2014dy@163.com (L.Z.); Linaxu_632@126.com (L.X.); taoxufengdalian@163.com (X.T.); Xuhan2002zs@163.com (X.H.); Lianhongyin_1980@163.com (L.Y.)

**Keywords:** *Rosa laevigata* Michx, total flavonoids, renal ischemia reperfusion, oxidative stress, inflammation

## Abstract

Renal ischemia-reperfusion injury (IRI) is a major cause of acute kidney injury (AKI). Our previous studies have shown that the total flavonoids (TFs) from *Rosa laevigata* Michx fruit has various activities, however, there were no papers reporting the role of the TFs against renal IRI. In the present work, a hypoxia/reoxygenation (H/R) model in NRK-52E cells and ischemia-reperfusion model in rats were used. The results showed that the TFs significantly attenuated cell injury and markedly decreased serum creatinine (Cr) and blood urea nitrogen (BUN) levels in rats. Further investigation revealed that the TFs markedly decreased the levels of malondialdehyde (MDA), superoxide dismutase (SOD), glutathione (GSH) and glutathione peroxidase (GSH-Px) and intracellular reactive oxygen species (ROS), up-regulated the levels of silent information regulator factor 2-related enzyme 1 (Sirt1), nuclear factor erythroid 2-related factor-2 (Nrf2) and heme oxygenase-1 (HO-1), down-regulated the levels of Kelch like ECH-associated protein-1 (Keap1) and the nuclear translocation of nuclear factor-κBp65 (NF-κBp65), and decreased the mRNA levels of interleukine-1β (IL-1β), interleukin-6 (IL-6) and tumor necrosis factor-α (TNF-α). Furthermore, inhibiting Sirt1 by siRNA showed that the role of the natural product in protecting renal IRI was significantly attenuated, suggesting that the effect of the extract against renal IRI depended on Sirt1. Taken together, the TFs has significantly nephroprotective effect against IRI by affecting Sirt1/Nrf2/NF-κB signaling pathway, which should be developed as a new therapeutic agent or food additives to treat acute kidney injury in the future.

## 1. Introduction

Acute kidney injury (AKI), a common severe clinical syndrome, occurs in many clinical situations including kidney transplants, partial nephrectomy, heart surgery and sepsis. There are approximately 13.3 million affected people worldwide and about 2 million die from AKI every year [1,2,3,4]. Renal ischemia-reperfusion injury (IRI), a common cause of AKI, refers to the serious renal injury after ischemia-reperfusion (I/R), which has some complicated pathophysiological features including renal tubular cell necrosis, extracellular matrix (ECM) degradation and interstitial inflammatory cell infiltration. Furthermore, renal IRI can lead to the delayed recovery of renal function and acute rejection after transplantation. Therefore, it is necessary to explore effective therapeutic methods or new drugs to treat IRI. Nowadays, the underlying mechanisms of renal IRI are still not well understood. However, recent studies have shown that oxidative stress, inflammation and apoptosis are associated with the disease [5,6,7,8,9,10,11,12]. I/R-induced high levels of reactive oxygen species (ROS) and pro-inflammatory cytokines can cause lipid peroxidation and lead to renal cell death. Silent information regulator factor 2-related enzyme 1 (Sirt1), a member of deacetylation enzymes, can deacetylate various transcription factors including nuclear factor erythroid 2-related factor (Nrf2) and nuclear factor-κB (NF-κB), and affect crucial cellular pathways associated with oxidative stress and inflammation [13,14,15,16,17]. Nrf2, a transcription factor, combines with Kelch like ECH-associated protein 1 (Keap1) in cytoplasm under resting state [18,19,20,21]. Sirt1 can release Nrf2 by changing Keap1 conformation, and then Nrf2 translocates into the nucleus and binds with antioxidative response elements (ARE) to regulate various antioxidative genes against oxidative stress [17,22,23]. Furthermore, Sirt1 can also inhibit the transcriptional activity of NF-κB by interacting with p65 subunit, and reduce the levels of pro-inflammatory cytokines [13,24,25,26]. Therefore, up-regulating Sirt1 to suppress oxidative stress and inflammation represents a potentially nephroprotective treatment strategy [27].

Currently, some drugs including diuretics, erythropoietin and atorvastatin have been used to treat renal IRI [28]. However, they cannot play excellently preventive effects against renal injury in clinical [29]. Traditional Chinese medicines have been used in China to treat diseases for thousands of years, and some active natural products including dioscin, tea polyphenols, osthole and baicalein have been reported to protect against renal IRI [7,30,31,32]. Thus, it is reasonable to seek effective natural products from medicinal herbs for the treatment of renal IRI.

*Rosa laevigata* Michx, belonging to the Rosaceae family, is mainly distributed in some countries in Asia, especially in the southeast and southwest of China. It is a well-known traditional Chinese medicine and often used to stop diarrhea and cure frequent micturition. Some researchers have shown that the fruit has the protective effects against diabetes and chronic cough caused by multiple chemicals including polysaccharose, flavonoids, saponins and triterpenes. Chemicals including ursolic acid, oleanolic acid, β-sitosterol, daucosterol, hederagenin and 2a,3b,19a-trihydroxyolean-12-en-28-oic acid are the main saponins from the fruit [33]. In our previous reports, the contents of some flavonoids, including quercetin, kaempferide and isorhamnetin have been determined in the total flavonoids (TFs) extracted from the fruit [34]. Furthermore, our previous studies have shown that the TFs fraction has antioxidant, anti-inflammatory, hypolipidemic and hepatoprotective activities [35,36,37,38]. However, there are no papers reporting the role of the TFs against renal IRI to the best of our knowledge. Thus, the aim of the present study was to investigate the effects and possible mechanisms of the natural product against renal IRI.

## 2. Results

### 2.1. TFs Protects NRK-52E Cells from H/R Injury

**Figure 1:**
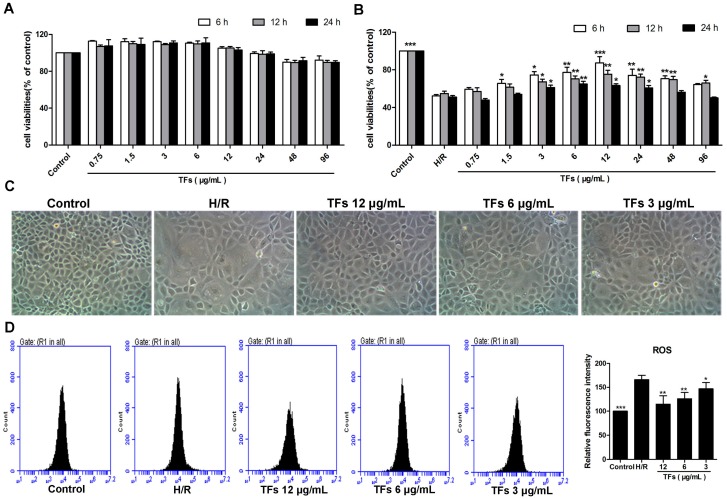
Effects of the TFs on protecting NRK-52E cells from H/R injury. (**A**) Effects of the TFs on the viabilities of NRK-52E cells; (**B**) Effects of the TFs on the viabilities of NRK-52E cells against H/R injury; (**C**) Effects of 6 h treatment with TFs (12, 6 and 3 μg/mL) on the cellular morphology and structure of NRK-52E cells by bright image (×100 magnification); (**D**) Effects of the TFs (12, 6 and 3 μg/mL) for 6 h treatment on the levels of intracellular ROS. Data are presented as the mean ±SD (*n* = 6). * *p* < 0.05, ** *p* < 0.01 and *** *p* < 0.001 compared with H/R group.

As shown in Figure 1A, treatment with the TFs at the concentrations of 0.75–96 μg/mL for 6, 12 or 24 h did not significantly affect NRK-52E (renal tubular duct epithelial cells of rat) cell viability. As shown in Figure 1B, pretreatment with the TFs at the concentration of 1.5–48 μg/mL for 6 h showed obviously protective effect against cell injury. In addition, compared with model group, the crude extract at the concentrations of 3, 6 and 12 μg/mL for 6 h significantly increased cell viabilities by 1.42-, 1.48- and 1.67-fold. Therefore, the natural product at the concentrations of 3, 6 and 12 μg/mL for 6 h was selected to protect NRK-52E cells against H/R injury. As shown in Figure 1C, the morphological changes of the cells caused by H/R were significantly restored by the TFs. These results suggested that the TFs showed protective effect against NRK-52E cells from H/R injury.

### 2.2. TFs Reduces ROS Levels in NRK-52E Cells

As shown in Figure 1D, the levels of intracellular ROS were markedly decreased in the TFs-treated groups compared with H/R group, indicating that the TFs attenuated H/R-caused oxidative stress in NRK-52E cells.

### 2.3. TFs Improves Renal I/R Injury in Rats 

**Figure 2:**
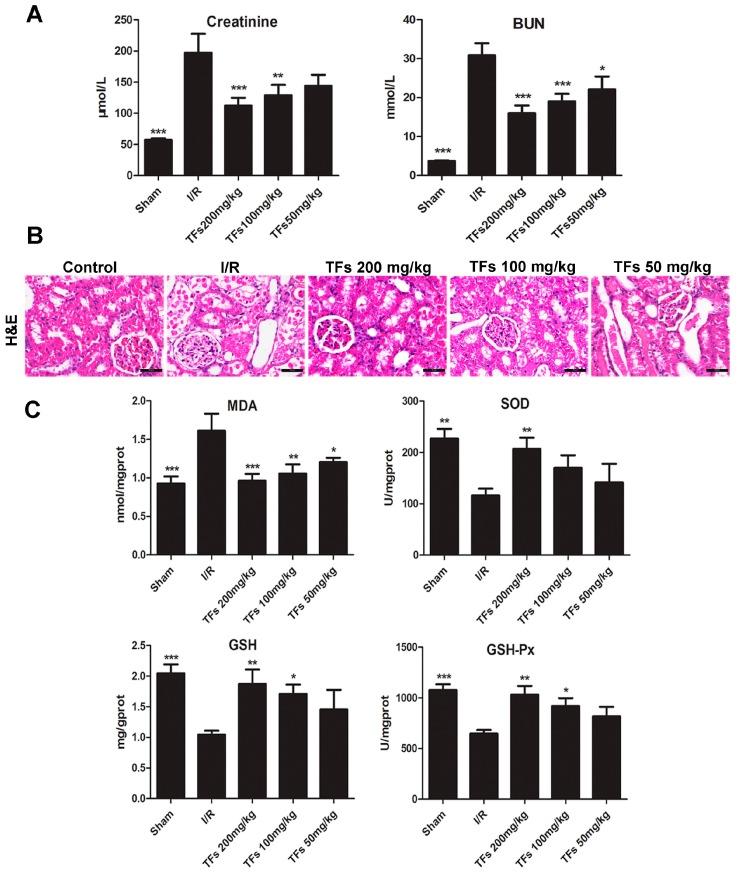
Effects of the TFs on renal I/R injury in rats. (**A**) Effects of the TFs on serum Cr and BUN levels after renal I/R injury; (**B**) Hematoxylin and Eosin (H&E) staining of kidney tissues (magnification, 200×); (**C**) Effects of the TFs on the levels of MDA, SDO, GSH, GSH-Px in renal tissues. Data are presented as the mean ±SD (*n* = 6–10). * *p* < 0.05, ** *p* < 0.01 and *** *p* < 0.001 compared with I/R group.

We detected the serum markers of renal injury using commercial kits. As shown in Figure 2A, the levels of serum creatinine (Cr) and blood urea nitrogen (BUN) in I/R group were obviously increased from 57.4 ± 6.7 to 197.4 ± 85.4 μmol/L and from 3.7 ± 0.4 to 30.8 ± 9.8 mmol/L, respectively, compared with control group, which were significantly restored by the TFs.

As shown in Figure 2B, H&E staining indicated that the kidney tissues in I/R group exhibited extensive swelling in renal tubular epithelial cells, vacuole degeneration, disappearance of brush border, coagulation necrosis, and the increased inflammatory cell numbers, which were markedly restored by the TFs.

### 2.4. TFs Attenuates Oxidative Stress in Vivo

As shown in Figure 2C, the renal tissue levels of superoxide dismutase (SOD, 116.1 ± 32.9 U/mg prot), glutathione (GSH, 1.0 ± 0.2 mg/g prot) and glutathione peroxidase (GSH-Px, 647.9 ± 86.6 U/mg prot) in I/R group were markedly decreased, and the malondialdehyde (MDA) level (1.6 ± 0.4 nmol/mg prot) was significantly increased compared with sham group, which were all reversed by the TFs. These results indicated that the TFs attenuated I/R-induced oxidative stress in vivo.

### 2.5. TFs Activates Sirt1/Nrf2 Signaling Pathway in Vitro and Vivo

**Figure 3:**
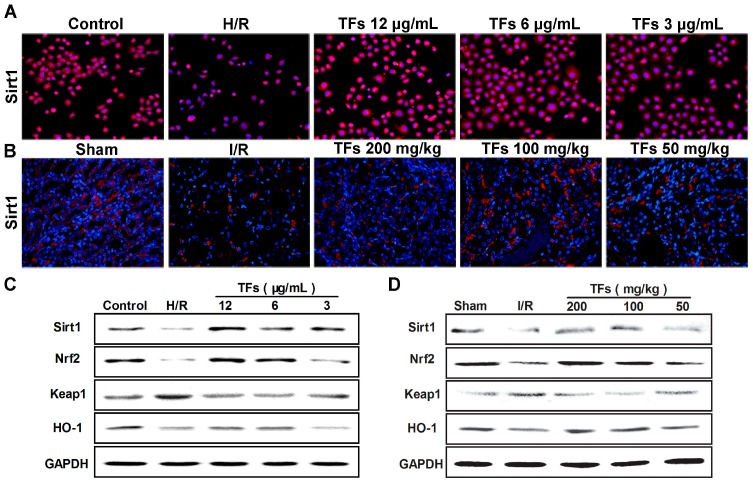
Effect of the TFs on Sirt1/Nrf2 signaling pathway. (**A**,**B**) Effects of the TFs on Sirt1 levels based on immunofluorescent assay (magnification, 200×) in NRK-52E cells and renal tissues; (**C**,**D**) Effects of the TFs on the protein levels of Sirt1, Nrf2, HO-1 and Keap1 in NRK-52E cells and renal tissues using western blotting assay.

As shown in Figure 3A,B, compared with model groups (H/R model group in vitro and I/R model group in vivo), the TFs significantly increased Sirt1 levels in NRK-52E cells and renal tissues based on immunofluorescence assay (magnification, 200×). Moreover, as shown in Figure 3C,D, compare with model groups, the natural product significantly up-regulated the levels of Sirt1, Nrf2, HO-1, and down-regulated the level of Keap1 in NRK-52E cells and renal tissues based on western blotting assay.

### 2.6. TFs Attenuates Inflammation in Vitro and Vivo

**Figure 4:**
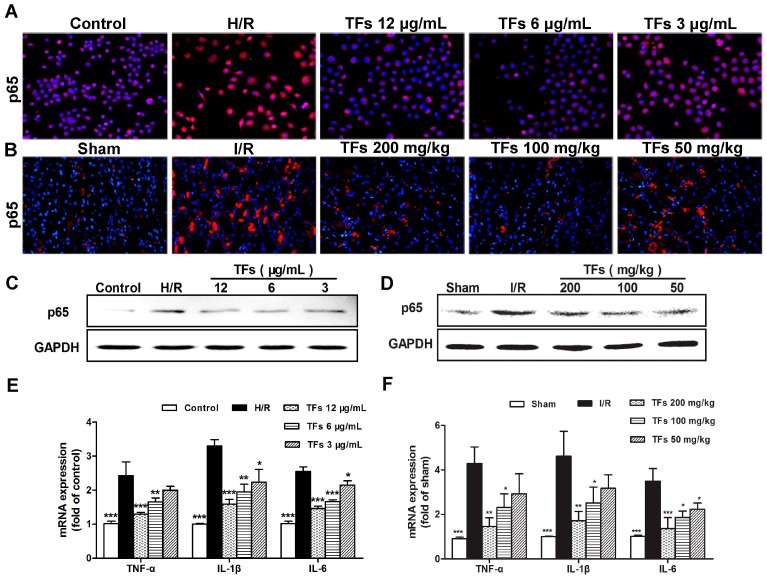
Effect of the TFs on inflammation. (**A**,**B**) Effects of the TFs on NF-κBp65 levels based on immunofluorescent assay (magnification, 200×) in NRK-52E cells and renal tissues; (**C**,**D**) Effects of the TFs on the protein levels of NF-κBp65 in NRK-52E cells and renal tissues using western blotting assay; (**E**,**F**) Effects of the TFs on the levels of TNF-α, IL-1β and IL-6 in NRK-52E cells and renal tissues using real-time PCR assay. Data are presented as the mean ±SD (*n* = 3–6). * *p* < 0.05, ** *p* < 0.01 and *** *p* < 0.001 compared with model groups (H/R model group or I/R model group).

As shown in Figure 4A–D, the expression levels of NF-κBp65 in model groups were markedly increased compared with control groups using immunofluorescencet (magnification, 200×) and western blotting assays in NRK-52E cells and renal tissues, which were obviously reversed by the TFs. In addition, as shown in Figure 4E,F, the mRNA levels of interleukine-1β (IL-1β), interleukin-6 (IL-6) and tumor necrosis factor-α (TNF-α) in NRK-52E cells and renal tissues were markedly increased in model groups, which were all restored by the TFs. These results indicated that TFs attenuated H/R-induced inflammation in vitro and in vivo.

### 2.7. Sirt1 siRNA Abrogates the Nephroprotective Effects of the TFs

**Figure 5:**
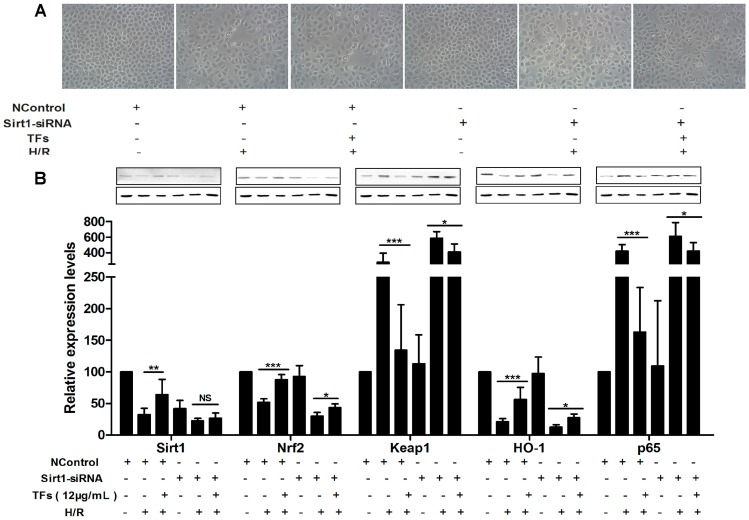
Sirt1 siRNA reversed the effects of the TFs against renal IRI. (**A**) Effects of the TFs after Sirt1 siRNA transfection on the cellular morphology and structure of NRK-52E cells by bright image (×100 magnification). (**B**) Effects of the TFs on the protein levels of Sirt1, Nrf2, HO-1, Keap1 and NF-κBp65 after Sirt1 siRNA transfection in NRK-52E cells. Data are presented as the mean ±SD (*n* = 3). * *p* < 0.05, ** *p* < 0.01 and *** *p*< 0.001; NS, not significant.

As shown in Figure 5A, the morphological changes of NRK-52E cells caused by H/R were restored by the TFs (12 μg/mL), whereas the protective effect of the extract was slightly reduced after inhibiting Sirt1. As shown in Figure 5B, the levels of Sirt1, Nrf2 and HO-1 were decreased, and the levels of Keap1 and NF-κBp65 were increased in H/R + Sirt1 siRNA group compare with H/R group, indicating that H/R-induced injury was more serious after inhibiting Sirt1. In addition, the role of the TFs on protecting renal IRI was significantly attenuated in TFs + Sirt1 siRNA group. Altogether, inhibiting Sirt1 suggested that the effect of the TFs against renal IRI depended on Sirt1.

## 3. Discussion

AKI, associated with high morbidity and mortality, can increase the development of chronic kidney disease and progression to end-stage kidney disease. Renal IRI is not only a common cause of AKI, but also an important problem affecting the recovery of renal function after transplantation. Furthermore, it can lead to the prolonged hospitalization or even organ loss of patients in clinical. Currently, no effective therapeutic agents or methods are available for the treatment of renal IRI [39]. In this study, the protective effect of the TFs against renal IRI was investigated on hypoxia/reoxygenation model in NRK-52E cells at first. The results showed that pretreatment with the TFs significantly attenuated cell injury and histological damage, suggesting that the natural product showed protective effect against renal IRI in vitro. Then, we tested the effect of the TFs on renal I/R injury in rats, and the results of serum markers and histological examination suggested the natural product showed protective effect against renal IRI in vivo.

Many studies have suggested that oxidative stress and inflammation are involved in I/R-induced acute kidney injury. Excess generation of ROS and pro-inflammatory cytokines can cause severe cellular and tissue injury through inducing oxidative damage of biological macromolecules and inflammatory cell infiltration [40,41]. Thus, the method to ameliorate oxidative stress and inflammation is promising to prevent IRI. The natural product, the TFs extracted from *R. laevigata* Michx exhibited powerful antioxidant action by enhancing antioxidant enzyme activities in vitro in our previous works [35,42]. What’s more, the TFs has the protective effect against cerebral I/R injury through suppression of inflammation [43]. Our results in the present study showed that the TFs markedly decreased the levels of intracellular ROS, the oxidative stress-related biomarkers, and the mRNA levels of IL-1β, IL-6 and TNF-α, indicating that the TFs prevented oxidative stress and inflammation against renal IRI. 

Sirt1 can regulate many physiological functions, including oxidative stress and inflammation, which are the key processes during ischemia/reperfusion injury [13]. Previous studies have shown that Sirt1 can counter cerebral IRI through anti-inflammatory and anti-apoptotic mechanism. Resveratrol, another class of polyphenol, has been shown to play the protective role in kidney disease through up-regulating Sirt1 level [44].

Transcription factor Nrf2 is a master regulator of the cellular antioxidant response through Nrf2/Keap1 signaling pathway. Sirt1 can activate Nrf2 by release of Keap1 from Nrf2-Keap1 complex, and then Nrf2 translocates into the nucleus to regulate various antioxidative genes against oxidative stress [45,46]. NF-κB is well-known as an inflammation promoting transcription factor that contributes to immune cell infiltration and cytokine production in many diseases. Sirt1 can abolish NF-κB activation by interacting with p65 subunit and then block the process of inflammation [47]. In the present study, we found that the TFs significantly increased the levels of Sirt1, Nrf2 and HO-1, decreased the levels of Keap1 and NF-κBp65. Moreover, depletion of Sirt1 markedly attenuated the protective effect of the TFs in vitro by affecting the transcriptional activities of Nrf2 and NF-κBp65. Taken together, these findings suggested that the TFs reduced renal oxidative stress and inflammation against renal IRI through Sirt1/Nrf2/NF-κB signaling pathway.

These findings provide novel insights into the mechanism of the TFs extracted from *R. laevigata* Michx as a candidate for the treatment of renal IRI in the future. However, the underlying mechanisms are more complex than what is described here, and our results do not exclude the possible involvement of other mechanisms caused by the TFs to treat renal I/R injury. Obviously, future studies are needed to investigate its efficacy and mechanisms, in order to provide materials for successfully translated into clinical use.

## 4. Materials and Methods

### 4.1. Materials

Creatinine (Cr), blood urea nitrogen (BUN), malondialdehyde (MDA), superoxide dismutase (SOD), glutathione (GSH) and glutathione peroxidase (GSH-Px) detection kits were purchased from Nanjing Jiancheng Institute of Biotechnology (Nanjing, China). Enhanced Bicinchoninic Acid (BCA) Protein Assay Kit, Reactive Oxygen Species Assay Kit, Cell lysis buffer for Western and IP and phenylmethanesulfonyl fluoride (PMSF) were obtained from Beyotime Institute of Biotechnology (Jiangsu, China). Sodium dodecyl sulfate (SDS), hydroxymethyl aminomethane (Tris) and 4′,6′-Diamidino-2-phenylindole (DAPI) were purchased from Sigma (St. Louis, MO, USA). 3-(4,5-Dimethylthiazol-2-yl)-2,5-diphenyl tetrazolium bromide (MTT) was provided by Roche Diagnostics (Basel, Switzerland). Hematoxylin, 2-step plus^®^Poly-HRP Anti-Mouse/Rabbit IgG Detection System and 3,3′-Diaminobenzidine tetrahydrochloride (DAB) Substrate Kit were provided by Zhongshan Golden Bridge Biotechnology (Beijing, China). TransZol™, TransScript^®^ All-in-One First-Strand cDNA Synthesis SuperMix for qPCR(One-Step gDNA Removal) and TransStart^®^ Top Green qPCR SuperMix were supplied by Beijing TransGen Biotech Co., Ltd. (Beijing, China). Sirt1 siRNA (Sequence: CCACCTGAGTTGGATGATA) was purchased from Guangzhou RiboBio Co., Ltd. (Guangzhou, China), and Lipofectamine 2000 was obtained from GenePharma (Shanghai, China).

### 4.2. Herbal Material and the Total Flavonoids

The fruit of *R. laevigata* Michx was purchased from Yunnan Qiancaoyuan Pharmaceutical Company Co. Ltd. (Yunnan, China) and identified by Dr. Yunpeng Diao (College of Pharmacy, Dalian Medical University, Dalian, China). A voucher specimen (DLMU, JYZ080426) was deposited in the Herbarium of the College of Pharmacy of Dalian Medicinal University. The total flavonoids (TFs) was obtained from the fruit using the method described in our previous study [34]. Briefly, the dried *R. laevigata* Michx fruit was crushed and extracted with 60% aqueous ethanol (sample:solvent = 1:8, *w*/*v*) for two times (two hours for each) under heat and reflux. After condensation under low pressure at 60 °C, the extracted solution was added to a D101 macroporous resin (Chemical Plant of Nankai University, Tianjin, China) column. After elution with water, the fraction eluted with 40% aqueous ethanol was collected and evaporated under low pressure at 60 °C to dryness, and the dry powders were preserved at 4 °C for subsequent experiments. The content of the TFs in the crude extract was determined to be 81.3% by the colorimetric method described in our previous study [36]. The crude extract was dissolved in saline for in vivo experiments and in phosphate buffer saline (PBS) for in vitro experiments.

### 4.3. Cell Culture 

NRK-52E cells were purchased from the Shanghai Institutes for Biological Sciences (Shanghai, China), and cultured at 37 °C in an atmosphere with 5% CO_2_ and 95% O_2_ in DMEM containing 10% fetal bovine serum (FBS). Cells in the H/R model group were cultured for 6 h under hypoxic conditions (1% O_2_, 94% N_2_, and 5% CO_2_) provided by an incubator in medium without nutrients (1.13 mM CaCl_2_, 5.0 mM KCl, 0.3 mM KH_2_PO_4_, 0.5 mM MgCl_2_·6H_2_O, 0.4 mM MgSO_4_·7H_2_O, 128 mM NaCl, 4 mM NaHCO_3_ and 10 mM HEPES), and then returned to a regular condition (5% CO_2_ and 95% air) for 12 h in serum-free medium for reoxygenation. Cells in TFs-treated groups were pretreated with the TFs (3, 6 and 12 μg/mL) for 6 h before H/R operation. Meanwhile, the serum of the medium in control group was removed.

### 4.4. Cell Viability Assay

The NRK-52E cells were plated in 96-well culture plates at a density of 5 × 10^4^ cells/mL, and incubated at 37 °C, 5% CO_2_ until approximately 70% confluence. The medium was replaced with serum-free DMEM containing various concentrations (0.75, 1.5, 3, 6, 12, 24, 48 and 96 μg/mL) of TFs for 6, 12 and 24 h before H/R at 37 °C. At the end of H/R, MTT (10 μL, 5 mg/mL) solution was added to each well. After incubation at 37 °C for 4 h, the medium with MTT was removed. Next, 150 μL of DMSO was added to each well to dissolve the formazan crystals, and the absorbance at 490 nm was measured using a POLARstar OPTIMA multi-detection microplate reader (BioRad, San Diego, CA, USA).

### 4.5. Measurement of Intracellular ROS

The NRK-52E cells were plated in 6-well culture plates at the density of 5 × 10^4^ cells/mL and treated with the TFs at the concentrations of 3, 6 and 12 μg/mL for 6 h before H/R. The cells were harvested and then re-suspended in 1 mL DCFH-DA (10.0 μM) for the detection of ROS, which was analyzed by flow cytometry (Becton-Dickinson, Lake Franklin, NJ, USA).

### 4.6. Animals

Fifty Male SD rats, weighing 180–220 g, were obtained from the Experimental Animal Center of Dalian Medical University (Dalian, China). All animals had free access to food and water during the experiments. All experimental procedures were approved by the Animal Care and Use Committee of Dalian Medical University and performed in strict accordance with the People’s Republic of China Legislation Regarding the Use and Care of Laboratory Animals. Our previous work has shown that the TFs at the doses of 50, 100 and 200 mg/kg in rats has good antioxidant and anti-inflammatory effects against cerebral ischemia/reperfusion injury [43]. Thus, in the present study, the doses of the crude extract in rats were set at 50, 100 and 200 mg/kg. Then, the animals were randomly divided into five groups: sham, I/R (model group), TFs 200 mg/kg + I/R, TFs 100 mg/kg + I/R and TFs 50 mg/kg + I/R groups. The crude extract (dissolved in saline) was intragastrically administered at the doses of 50, 100 and 200 mg/kg once daily for seven consecutive days before surgery. Meanwhile the rats in sham and I/R groups were administered with saline. All rats were anesthetized with an intraperitoneal injection of 10% chloral hydrate (3.5 mL/kg) and placed on a heating pad to maintain body temperature at 37 °C. Both renal pedicles were identified, and clamped for 45 min followed by reperfusion for 24 h except the rats in sham group. All rats were sacrificed after recovery of 24 h, and the blood and kidney tissue samples were collected for subsequent experiments.

### 4.7. Measurement of BUN, Cr, MDA, SOD, GSH and GSH-Px Levels

The blood samples were centrifuged at 3500 r/min for 15 min, and the supernatant fluid was produced to detect the serum levels of Cr and BUN using Commercial kits according to the instructions. The kidney tissues were placed in cold saline (1:10, *w*/*v*), homogenized with a homogenizer machine, and then centrifuged at 3000 r/min for 15 min to produce the supernatant fluid for detecting the levels of MDA, SOD, GSH and GSH-Px using commercial kits according to the respective instructions.

### 4.8. Histopathologic and Immunofluorescence Assays

The kidney tissues were fixed in 10% formalin and embedded in paraffin, and the 5-μm-thick sections were stained with hematoxylin-eosin (H&E). Immunofluorescence staining of tissue slices or formalin-fixed cells for Sirt1 and p65 was performed using anti-Sirt1 and anti-p65 antibodies in a humidified chamber at 4 °C overnight, followed by incubation with an Alexa fluorescein-labeled secondary antibody at 37 °C for 1 h. Cell nuclear were stained with DAPI (5 μg/mL). All samples were analyzed by fluorescence microscopy (Olympus, Tokyo, Japan) at 200× magnification.

### 4.9. Real-Time PCR Assay

Total RNA samples were obtained from kidney tissues and NRK-52E cells using TransZol™ reagent following the manufacturer’s protocol. After purity determination, each RNA sample was reverse transcribed into cDNA using TransScript^®^ All-in-One First-Strand cDNA Synthesis SuperMix for qPCR(One-Step gDNA Removal) Kit. The mRNA levels were quantified by real-time PCR with TransStart^®^ Top Green qPCR SuperMix kit in an ABI 7500 Real-Time PCR System (Applied Biosystems, Carlsbad, CA, USA). The Ct value of the target genes was normalized to that of GAPDH. The unknown template in our study was calculated through the standard curve for quantitative analysis. The primers used in our work are given in Table 1.

### 4.10. Western Blotting Assay

Total protein samples were extracted from kidney tissues and NRK-52E cells using appropriate cold lysis buffer containing 1 mM PMSF following the manufacturer’s instructions. After determination of the contents, the proteins were separated by SDS-PAGE (8%–12%), transferred to PVDF membranes (Millipore, MA, USA), then blocked with 5% dried skim milk (Boster Biological Technology, Wuhan, China) and incubated overnight at 4 °C with the primary antibodies listed in Table 2. The membranes were incubated with an HRP-conjugated secondary antibody for 2 h at room temperature. And protein detection was performed based on an enhanced chemilumines-cence (ECL) method and photographed by using a Bio-Spectrum Gel Imaging System (UVP, Upland, CA, USA). Intensity values of the relative protein levels were normalized to GAPDH.

### 4.11. Sirt1 siRNA Transfection

The NRK-52E cells were plated in six-well plates at a density of 2 × 10^5^ cells/mL, incubated until approximately to 70% confluence, and then transfected with control siRNA or Sirt1 SiRNA using lipofectamine 2000 reagent according to the manufacturer’s instructions. Briefly, lipofectamine 2000 reagent was dissolved in reduced Serum Media (Opti-MEM) and then equilibrated for 5 min at room temperature to obtained solution A. Meanwhile, the sirt1-siRNA and negative control siRNA were also dissolved in Opti-MEM as solution B. Next, the solutions A and B were mixed, and tequilibrated for 20 min at room temperature to form a mixture of siRNA-lipo2000. The cells were transfected with sirt1-siRNA or negative control siRNA through interaction with the mixture. After 6 h of transfection, the cells were treated with the TFs (12.0 μg/mL) for 6 h before H/R, and then the protein levels of Sirt1, Nrf2, Keap1, HO-1 and NF-κBp65 were determined. 

### 4.12. Statistical Analysis

All results are expressed as the mean ± standard deviation (SD), and analyzed by one-way analysis of variance (ANOVA) coupled with LSD in Post Hoc Multiple Comparisons using the SPSS 17.0 statistical software. *p*-Values less than 0.05 were considered to be statistically significant.

## 5. Conclusions

In summary, in this study, we demonstrated that the TFs from *R. laevigata* Michx fruit has protective effect against renal I/R injury through attenuating oxidative stress and inflammation for the first time, which should be developed as a new therapeutic agent or food additives to treat acute kidney injury in the future.

## Figures and Tables

**Table 1:** The primer sequences used for real-time PCR assay in rats.

Gene	GenBank	Forward Primer (5′–3′)	Reverse Primer (5′–3′)
GAPDH	NM_017008.3	GGCACAGTCAAGGCTGAGAATG	ATGGTGGTGAAGACGCCAGTA
IL-1β	NM_031512.2	GACTTCACCATGGAACCCGT	CGAGACTGCCCATTCTCGAC
IL-6	NM_012589.1	ATTGTATGAACAGCGATGATGCAC	CCAGGTAGAAACGGAACTCCAGA
TNF-α	NM_012675.3	TCAGTTCCATGGCCCAGAC	GTTGTCTTTGAGATCCATGCCATT

**Table 2:** The antibodies used for western blotting assays.

Primary Antibody *	Source	Dilution
Sirt1	Rabbit	1:1000
Nrf2	Rabbit	1:1000
Keap1	Rabbit	1:1000
HO-1	Rabbit	1:1000
p65	Rabbit	1:1000
GAPDH	Rabbit	1:1000

* Source: Proteintech Group, Chicago, IL, USA.
