# Correction: Zhao, L.S., et al. Protective Effect of the Total Flavonoids from *Rosa laevigata* Michx Fruit on Renal Ischemia–Reperfusion Injury through Suppression of Oxidative Stress and Inflammation. *Molecules* 2016, *21*, 952.

**DOI:** 10.3390/molecules24244628

**Published:** 2019-12-17

**Authors:** Lisha Zhao, Lina Xu, Xufeng Tao, Xu Han, Lianhong Yin, Yan Qi, Jinyong Peng

**Affiliations:** College of Pharmacy, Dalian Medical University, Western 9 Lvshunnan Road, Dalian 116044, China; lihua2014dy@163.com (L.Z.); Linaxu0112@163.com (L.X.); taoxufengdalian@163.com (X.T.); Xuhan0118@163.com (X.H.); Lianhongyin0112@163.com (L.Y.)

During the course of a review of our publications, an error in the title paper [1] has come to our attention. This error affects the cellular morphology and structure by bright image microscopy data presented in Figure 5A. We provide, below, the correct figure. The data has been reanalyzed and determined to have no influence on the reported results.

**Figure 5:**
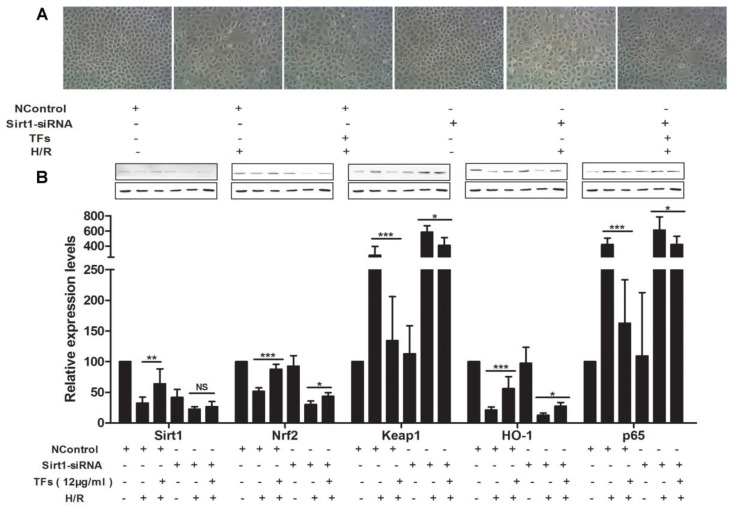
Sirt1 siRNA reversed the effects of the TFs against renal IRI. (**A**) Effects of the TFs after Sirt1 siRNA transfection on the cellular morphology and structure of NRK-52E cells by bright image (×100 magnification). (**B**) Effects of the TFs on the protein levels of Sirt1, Nrf2, HO-1, Keap1 and NF-κBp65 after Sirt1 siRNA transfection in NRK-52E cells. Data are presented as the mean ± SD (*n* = 3). * *p* < 0.05, ** *p* < 0.01 and *** *p* < 0.001; NS, not significant.

All co-authors agree with the content of this Correction and wish to apologize for any inconvenience to the readers resulting from this error.
